# Associations of out of school physical activity, sedentary lifestyle and socioeconomic status with weight status and adiposity of Cameroon children

**DOI:** 10.1186/s40608-017-0171-3

**Published:** 2017-11-07

**Authors:** Lifoter K. Navti, Mary B. Atanga, Loveline L. Niba

**Affiliations:** 1 Department of Biochemistry, Catholic University of Cameroon (CATUC), Bamenda, P.O. Box 782, Bamenda, Cameroon; 2 Nutrition and Health Research Group (NHRG), Bamenda, Cameroon; 3 Department of Nursing and Midwifery, University of Bamenda, P.O. Box 39, Bambili, Bamenda, Cameroon

**Keywords:** Physical activity, Sedentary lifestyle, Overweight/obesity, Triceps skinfold thickness, Quantile regression

## Abstract

**Background:**

Low physical activity and a sedentary lifestyle are contributing to overweight/obesity in children. This study aims to explore relationships between out of school physical activity, sedentary lifestyle and socioeconomic status indicators with children’s weight status and adiposity.

**Methods:**

Five hundred twenty-two children of ages 5 to 12 years were randomly selected in a school-based cross sectional study in Bamenda, Cameroon. Weight and height were measured and BMI calculated. These variables were standardized for age and gender. Socioeconomic variables and proxy measures of physical activity and sedentary lifestyle of children were reported by parents using a structured questionnaire. Bivariate and multivariable logistic regression was used to calculate odds ratios.

Quantile regression was used to compare median values of triceps skinfold thickness across the different factors.

**Results:**

In bivariate analysis, physical activity > 4 – 7 times/week was significantly (*p* = 0.010) associated with a lower prevalence (5.9%) of overweight/obesity. In multivariable analysis, physical activity > twice a week (OR 0.1, 95% CI 0.05 – 0.3), sedentary lifestyle > 3 h/day (OR 2.4, 95% CI 1.2 – 4.3) and being in the high occupation class (OR 4.3, 95% CI 2.2 – 8.1) independently predicted overweight/obesity. With quantile regression, physical activity > 4 – 7 times/week was significantly (*p* = 0.023) associated with a 1.36 mm decrease in median triceps skinfold thickness, while sedentary lifestyle (> 3 h/day) (*p* = 0.026) and being in the high occupation class (*p* = 0.007) were significantly associated with a 1.37 mm and 1.86 mm increase in median triceps skinfold thickness respectively.

**Conclusion:**

Physical activity is inversely related to BMI-defined overweight/obesity and triceps skinfold thickness. Also, a high sedentary lifestyle and a high occupation class were associated with overweight/obesity and had the largest significant relationship with triceps skinfold thickness. There is need to objectively assess physical activity and sedentary lifestyle in our setting, in and out of school. Also longitudinal studies are warranted to understand the influence of cultural and behavioral drivers of physical activity and sedentary lifestyle.

## Background

Overweight/obesity is affecting children in both developed and developing countries and had been described as a global threat to health more than a decade ago [1]. It affects approximately one-tenth of school-age children globally [2]. Increasing numbers in overweight/obese children have been recorded in developing countries undergoing an epidemiologic transition [3, 4], which is characterized by adoption of a sedentary lifestyle, physical inactivity, changes in dietary habits, rapid urbanization and better income [5–7].

Physical activity influences energy balance and has been shown to be beneficial to the health of school-age children [8, 9], with the likelihood of the children becoming more active in adulthood [10]. For instance, a follow-up study among US children between the ages of 10 and 15 years by Berkey et al. [11] revealed that when activity increased, BMI dropped. Also, lower levels of exercise has been shown to be associated with increased child adiposity [12, 13]. Despite the documented benefits of physical activity, evidence relating physical activity and overweight/obesity has not been consistent even in cohort studies, which indicated either weak or no associations between physical activity and overweight/obesity in children [14, 15].

Leisure time activities in children have changed over the past years as a result of advances in technology. In Cameroon, there is no documented evidence on leisure time activities in children. However, evidence from other countries indicate that children spend more time in sedentary activities like watching TV, internet and playing video games [16]. A report revealed that TV watching and playing of video games are common among younger children, while internet activities are common among adolescents, and a high proportion (> 80%) of the children spend on average, one and a half hours a day watching TV [17]. This is concerning because a recent study indicated that the use of electronic media has been associated with childhood overweight/obesity in school-age children [18]. However, an Australian study did not find such associations among adolescents [19].

In some African countries, less than half of children and youths meet up with the WHO daily requirement of 60 min of moderate-to-vigorous intensity physical activity [20]. Also, a study in Senegal indicated that adolescents occupy themselves with sedentary activities for half of their time [21]. The opportunities for physical activity are influenced by socioeconomic, cultural and even geographical factors, which contribute to differences in the numbers of individuals affected with overweight/obesity. For instance, a recent report in rural South Africa indicated that sedentary time and physical activity were independently predicted by socioeconomic status (SES) [22]. Other, studies had revealed that subjects from urban settings were least active compared to their rural peers [23, 24] and low physical activity level was associated with attending a private school [25]. However, most of these studies used body mass index (BMI), which does not directly reflect adiposity levels [26, 27].

The primary goal of this study is to investigate the contribution of selected factors (out of school physical activity, out of school sedentary lifestyle and SES indicators) to children’s weight status and adiposity, using body mass index and triceps skinfold thickness respectively.

## Methods

### Subjects

This school-based cross sectional analysis included a randomly selected sample of children (5 – 12 years) from 6 primary schools in Mezam Division of the North West Region (NWR) of Cameroon. A list of both private and public primary schools in Mezam Division of the NWR was obtained from the North West regional delegation for basic education. In order to have an adequate mix of socioeconomic groups and taking into consideration the distribution of more schools in urban than rural settings, 4 schools in the urban (2 private, 2 public) and 2 schools in the rural (1 private, 1 public) settings were randomly selected and contacted. Any school that refused to participate was replaced by another randomly selected one on the list. Based on the presumption that each class in schools should have a maximum of 40 - 45 children, 20 children were randomly selected from the 6 classes in each school. This was done to maximize the age distribution of the sample. A total of 720 children were randomly selected. Consent information which included the objectives of the study and also the study questionnaire were sent to parents/guardians through their children. The consent information was also given to the head teachers of the different schools. 609 questionnaires were returned giving a response rate of 84.6%. In addition, 87 participants were dropped because of missing data on physical activity, sedentary lifestyle and some SES indicators. However, there were no significant differences in anthropometric variables between those dropped and those retained in the study. A final sample of 522 children (262 boys and 260 girls) was included in the analysis.

### Ethical considerations

Ethical approval for this study was obtained from the institutional review board of the Catholic University of Cameroon (CATUC), Bamenda. Administrative clearances were obtained from the North West regional delegations for basic education and public health. All head teachers and parents/guardians gave written informed consent before the study commenced. Verbal assent was also obtained from each child before any study related procedure was carried out.

## Data collection

### Anthropometry

All measurement activities were carried out by trained nurses using standardized protocol while children were in school during morning hours. Body weight, to the nearest 0.1 kg of the children was obtained using a portable digital scale (Omron BF 511, Japan) in light clothing and without shoes. Height was measured to the nearest 0.1 cm using a portable stadiometer (Seca 213, Germany) without shoes. BMI, in kg/m^2^ was calculated from weight and height and used as a measure of the weight status of participants. Triceps skinfold thickness was measured half-way down and parallel to the long axis of the flexed left arm (between the acromion and olecranon) hanging on the child’s side using a standard skinfold caliper (Holtain Ltd., Croswell, Crymch, UK) to the nearest 0.2 mm that functioned under a constant pressure of 10 g/mm^2^ [28]. The skinfold measurements were carried out in triplicates by one observer (LKN) and the average value was recorded and used as a measure of adiposity. The children’s dates of birth and gender were obtained from school records during anthropometric measurements.

The information on school type (private or public) was obtained from the school administration.

### Parent/guardian questionnaire

Information on the children’s socioeconomic background, physical activity and sedentary lifestyle were reported by parents/guardians using a pre-tested questionnaire. This questionnaire was established in both English and French. In the absence of a validated instrument to assess activity levels of school-age children in our setting, the established physical activity and sedentary lifestyle components of our questionnaire were similar to that of a previous study in Pakistan [29]. However, no validity test was carried out. We adapted the questionnaire used in Pakistan in two ways. Firstly, instead of asking participants to indicate the frequency of participation in physical activity and sedentary lifestyle, our questionnaire provided categories of frequency of participation in both activities for participants to choose. Secondly, a sports activity that is not common among school children in our setting, like playing cricket, was substituted with football, which also involve physical movement.

The parents/guardians reported the out of school physical activity levels/sedentary lifestyle of their children and also provided information on their socioeconomic background (monthly income, professions and level of education) as explained below:

### Out of school physical activity

In this study, out of school physical activities included the following; farm work, household chores, ball dodging, hopscotch, skipping rope, hide and seek, dancing, football, handball, jogging, tennis, cycling, play on the playground and walking to school. The parents selected the frequency of the children’s involvement in each of the above activities in a week (Monday to Friday and Saturday to Sunday) [30] from the following categories: (i) one time/week, (ii) 2 times/week, (iii) 3 times/week, (iv) 4 times/week, (v) 5 times/week, (vi) 6 times/week and (vii) 7 times/week. The average of the number of times involved in all the activities was calculated and used to assign the children into the following groups; low (≤ 2 times/week), moderate (> 2 – 4 times/week) and high (> 4 – 7 times/week) physical activity.

### Out of school sedentary lifestyle

The out of school sedentary lifestyle of the children included; playing board games/cards, playing video games, reading/doing homework, watching TV, using the computer (internet), listening to music and motorization to school. The parents also selected the number of hours their children spend in the above activities during weekdays and weekend days from the following categories: (i) one hour/day, (ii) 2 h/day, (iii) 3 h/day, (iv) 4 h/day, (v) 5 h/day and (vi) 6 h/day. The mean time (in hours/day) [31] spent on all the activities was calculated and used to stratify participants as follows; low (≤ 1 h/day), moderate (> 1 – 3 h/day) and high (> 3 – 6 h/day) sedentary lifestyle.

No significant differences were observed in physical activity and sedentary lifestyle between weekdays and the weekend days.

### Socioeconomic status indicators

- *Occupation class*: Parents/guardians were asked to provide information on their monthly income and professions. The Cameroon public service classification system of civil servants based on income and profession was used to determine the occupation class of the parents. The civil servant categories A, B and C were used to classify parents/guardians into the high, middle and low occupation classes respectively [32]. Each child was designated to the parent in the highest occupational class.
- *Education level*: The education level of parents was also obtained from the questionnaire and they were categorized as follows; illiterate, primary, secondary and higher education after having spent none, ≤ 6 years, 7 to 13 years, and > 13 years of education respectively. The parent with the highest level of education was used to assign the children to the appropriate education group.

### Statistical analysis

The distribution of continuous variables was assessed using the Kolmogorov-Smirnov (K-S) test. Z-scores for weight, height and BMI were calculated using WHO AnthroPlus software, which utilizes the WHO 2007 growth reference data for children of ages 5 to 19 years [33]. A descriptive analysis was performed and values reported as mean (minimum – maximum) for continuous variables and proportions for categorized variables. Also, a parametric independent samples *t*-test and non-parametric Mann-Whitney *U* test were used to compare means of anthropometric variables between boys and girls, and proportions were compared using Chi square test.

Odds ratios, reported with their corresponding 95% confidence intervals for weight status (overweight/obesity), were calculated using bivariate and multivariable binary logistic regression models. The bivariate analysis included models which assessed the relationship between each factor and weight status. In multivariable analysis, odds ratios were adjusted for age, gender, design variables (school and class) and all the factors that were significant in the bivariate analysis. The design variables were included in the model in order to avoid violating the assumption of independence.

In addition, unadjusted analysis was carried out using the Kruskal-Wallis test to compare the median triceps skinfold thickness across the different factors. Further, the median triceps skinfold thickness across the different factors were adjusted using multiple quantile regression analysis. Quantile regression was used because it assesses the relationship across the distribution and the assumption of normality is not a requirement. The Powell Kernel approach for estimating standard errors [34] was used to calculate the regression estimates of the median of triceps skinfold thickness. The quantile regression analysis included all variables in the unadjusted analysis. The WHO criteria was used to define overweight (BMI z-score > +1) and obesity (BMI z-score > +2) [33] and statistical significance was considered at *p* < 0.05 level. The statistical software, R version 3.4.1 with QUANTREG package installed [34] was used for all statistical procedures.

## Results

The overall prevalence of overweight/obesity among the children in this study was 18.0%. When overweight and obesity were combined, more boys were affected than girls. However, this difference was not significant (*p* = 0.131) (Table 1). On the basis of the type of school attended, there was no significant difference in prevalence of overweight/obesity between children attending private schools (19.4%) and those in public schools (16.4%) (*p* = 0.330). Even though a higher proportion of urban subjects (18.4%) were overweight/obese compared to the rural subjects (17.1%), this difference was also not statistically significant (*p* = 0.775). In addition, Table 1 reveals that the mean BMI z-score was significantly higher for boys than girls (*p* < 0.001). On the contrary, mean triceps skinfold thickness was significantly higher for girls than boys (*p* = 0.018). No significant differences were observed for the other anthropometric variables.

**Table 1:** Summary characteristics of the study population by gender

Characteristics	Boys *N* = 262	Girls *N* = 260
Age (years)	9.1 (5.0 – 12.0)	8.9 (9.0 – 12.0)
Weight (kg)	30.8 (16.4 – 64.5)	30.8 (17.8 – 59.8)
Height (cm)	133.0 (104.6 – 176.1)	132.4 (109.2 – 161.2)
BMI (kg/m^2^)	17.1 (13.2 – 26.3)	17.2 (11.6 – 29.5)
Triceps skinfold (mm)	7.6 (3.0 – 23.5)^a^	10.2 (3.0 – 24.5)^a^
Weight z-score	0.22 (−2.81 – 3.60)	0.16 (−3.30 – 3.76)
Height z-score	−0.02 (−3.73 – 4.13)	0.05 (−3.91 – 6.19)
BMI z-score	0.39 (−2.20 – 2.88)^b^	0.20 (−3.62 – 3.61)^b^
BMI categories		
Severe thinness	0 (0.0)	2 (0.8)
Thinness	1 (0.4)	3 (1.2)
Normal weight	211 (80.5)	211 (81.2)
Overweight	45 (17.2)	33 (12.7)
Obese	5 (1.9)	11 (4.2)
Type of school		
Public	125 (47.7)	129 (49.6)
Private	137 (52.3)	131 (50.4)
Area of residence		
Urban	182 (69.5)	182 (70.0)
Rural	80 (30.5)	78 (30.0)

Values = mean (min – max) for continuous variables and n (%) for categorized variables

^a^ *p* < 0.001, ^b^ *p* < 0.05. BMI categories were defined using WHO criteria [33]

Table 2 shows the associations between the different factors with overweight/obesity as dependent variable. In bivariate analysis, moderate (> 2 – 4 times/week) (*p* < 0.001) and high (> 4 – 7 times/week) (*p* = 0.010) physical activities were significantly associated with a lower prevalence of overweight/obesity. Also, children in the middle (*p* < 0.001) and high (*p* < 0.001) occupation classes and those with a moderate (> 1 – 3 h/day) (*p* < 0.001) and high (> 3 – 6 h/day) (*p* = 0.012) sedentary lifestyle were significantly more likely to be overweight/obese compared to those in the low occupation class and those with a low sedentary lifestyle (≤ 1 h/day). Children attending private schools had higher odds of being overweight/obese when compared to their peers in public schools. However, this association was not significant. After controlling for age, gender, design variables (school and class) and all significant variables in the bivariate analysis (physical activity, sedentary lifestyle and occupation class), the multivariable analysis indicated that there was a significant independent inverse relationship between physical activity (> 2 – 4 times/week) (*p* < 0.001) and > 4 – 7 times/week (*p* = 0.010) with overweight/obesity. In addition, a significant independent positive association was observed between the high occupation class (*p* < 0.001) and high sedentary lifestyle (> 3 – 6 h/day) (*p* = 0.016) with overweight/obesity.

**Table 2:** Prevalence and odds ratios (95% CI) for the association of factors with weight status of school children (*N* = 522)

Factors	N	Overweight/obesity						
		Prevalence (%)	Bivariate OR	(95% CI)	*p*-value^a^	Multivariable OR	(95% CI)	*p*-value^a^
Physical activity					< 0.001			< 0.001
≤ 2 times/week	209	27.8	Ref.			Ref.		
> 2 – 4 times/week	194	14.9	0.2	(0.1 – 0.3)		0.1	(0.05 – 0.3)	
> 4 – 7 times/week	119	5.9	0.4	(0.1 – 0.8)		0.2	(0.1 – 0.6)	
Sedentary lifestyle					< 0.001			0.001
≤ 1 h/day	196	11.2	Ref.			Ref.		
> 1 – 3 h/day	203	17.2	3.1	(1.8 – 5.9)		3.3	(1.8 – 6.9)	
> 3 – 6 h/day	123	30.1	2.0	(1.2 – 3.4)		2.4	(1.2 – 4.3)	
Occupation class					< 0.001			< 0.001
Low	239	7.1	Ref.			Ref.		
Middle	149	14.8	9.7	(5.3 – 17.9)		8.6	(4.4 – 16.9)	
High	134	41.0	4.0	(2.3 – 7.1)		4.3	(2.2 – 8.1)	
Parental education					0.765			
Illiterate	129	20.2	Ref.					
Primary	107	15.9	0.8	(0.4 – 1.7)		–	–	–
Secondary	146	19.2	1.0	(0.5 – 2.0)		–	–	–
Higher education	140	16.4	0.8	(0.4 – 1.5)		–	–	–
Type of school					0.330			
Public	254	16.4	Ref.					
Private	268	19.4	1.2	(0.8 – 2.0)		–	–	–
Area of residence					0.774			
Rural	158	17.1	Ref.					
Urban	364	18.4	0.9	(0.6 – 1.5)		–	–	–

Multivariable odds ratios have been adjusted for age, gender, design variables (school and class), and all significant variables in bivariate analysis

*OR* Odds ratio, *CI* confidence interval. ^a^ *p*-value for whole variable significance

The unadjusted analysis in Table 3 shows the median of triceps skinfold thickness across the different factors. The overall median of triceps skinfold thickness was 8.0 mm. The median triceps skinfold thickness was significantly (*p* = 0.001) higher among children with low physical activity (≤ 2 times/week) compared to those with high physical activity (> 4 – 7 times/week). The factors that had the largest significant relationship with triceps skinfold thickness were sedentary lifestyle and occupation class: children in the high occupation class (*p* < 0.001) and those with a high sedentary lifestyle (> 3 – 6 h/day) (*p* < 0.001) had the highest median triceps skinfold thickness of 9.0 mm compared to 7.0 mm for those in the low occupation class and those with a low sedentary lifestyle (≤ 1 h/day). Also, the children in the urban area and those attending private schools had significantly higher median triceps skinfold thickness compared to their peers in the rural area and those attending public schools.

**Table 3:** Comparison of median of triceps skinfold thickness of school-age children (*N* = 522)

Factors	Triceps skinfold (mm)	
	Median	*p*-value^a^
Physical activity		0.001
≤ 2 times/week	8.0	
> 2 – 4 times/week	7.0	
> 4 – 7 times/week	7.0	
Sedentary lifestyle		< 0.001
≤ 1 h/day	7.0	
> 1 – 3 h/day	8.0	
> 3 – 6 h/day	9.0	
Occupation class		< 0.001
Low	7.0	
Middle	8.0	
High	9.0	
Parental education		0.625
Illiterate	7.8	
Primary	8.0	
Secondary	8.0	
Higher education	7.8	
Type of school		0.008
Public	7.0	
Private	8.0	
Area of residence		0.045
Rural	7.3	
Urban	8.0	

^a^Kruskal-Wallis test used to compare median triceps skinfolds across all groups. The 10th and 90th percentiles of triceps skinfold of all study participants were 3.6 and 16.0 mm respectively

Table 4 presents the findings of quantile regression estimates of the median of triceps skinfolds with their corresponding standard errors. A significant negative association was observed between high physical activity and triceps skinfold thickness (*p* = 0.023). The factors that had the largest significant relationship with triceps skinfold thickness were sedentary lifestyle: median triceps skinfold thickness of those with high sedentary activity (> 3 – 6 h/day) was 1.37 mm higher than those with a low sedentary activity (≤ 1 h/day) (*p* = 0.029); occupation class: those in the high occupation class had a 1.86 mm higher median triceps skinfold thickness than those from the low occupation class (*p* = 0.007).

**Table 4:** Multiple quantile regression estimates of the median of triceps skinfold thickness of school children (*N* = 522)

Factors	Median		
	Estimate	SE	*p*-value
Intercept	5.61	1.02	< 0.001
Physical activity			
≤ 2 times/week	0	–	–
> 2 – 4 times/week	−1.16	0.59	0.044
> 4 – 7 times/week	−1.36	0.60	0.023
Sedentary lifestyle			
≤ 1 h/day	0	–	–
> 1 – 3 h/day	1.22	0.44	0.006
> 3 – 6 h/day	1.37	0.62	0.029
Occupation class			
Low	0	–	–
Middle	0.57	0.47	0.224
High	1.86	0.68	0.007
Parental education			
Illiterate	0	–	–
Primary	0.34	0.68	0.619
Secondary	0.34	0.58	0.559
Higher education	−0.16	0.63	0.795
Type of school			
Public	0	–	–
Private	0.06	0.71	0.929
Area of residence			
Rural	0	–	–
Urban	−1.16	0.63	0.07

*SE* standard error. Age, gender and all factors were included in the quantile regression analysis. The estimate associated with each category is the difference in triceps skinfold thickness compared to the reference category

The differences in the proportions of physical activity and sedentary lifestyle with respect to occupation class are presented in Table 5. Both physical activity and sedentary lifestyle were significantly associated with occupation class. Also, low physical activity (≤ 2 times/week) and high sedentary lifestyle (> 3 – 6 h/day) were more common among children in the high occupation class. On the contrary, high physical activity (> 4 – 7 times/week) and low sedentary lifestyle (≤ 1 h/day) were more common among children in the low occupation class.

**Table 5:** The association of physical activity and sedentary lifestyle with occupation class (N = 522)

	Occupation class [%(95% CI)]						*p*-value^a^
	Low		Middle		High		
Physical activity							
≤ 2 times/week	37.2	(31.4 – 43.5)	38.9	(31.5 – 46.9)	46.3	(38.1 – 54.7)	0.016
> 2 – 4 times/week	35.1	(29.4 – 41.4)	40.3	(32.7 – 48.3)	37.3	(29.6 – 45.8)	
> 4 – 7 times/week	27.6	(22.3 – 33.6)	20.8	(15.1 – 28.0)	16.4	(11.1 – 23.6)	
Sedentary lifestyle							
≤ 1 h/day	43.1	(37.0 – 49.4)	38.9	(31.5 – 46.9)	26.1	(19.4 – 34.2)	0.001
> 1 – 3 h/day	36.8	(30.9 – 43.1)	39.6	(32.1 – 47.6)	41.8	(33.8 – 50.2)	
> 3 – 6 h/day	20.1	(15.5 – 25.6)	21.5	(15.6 – 28.7)	32.1	(24.8 – 40.4)	

^a^Chi square test. *CI* confidence interval

## Discussion

This study describes out of school physical activity and sedentary lifestyle of Cameroonian school-age children for the first time. The purpose of the study was to investigate whether there are relationships between out of school physical activity, sedentary lifestyle and socioeconomic status indicators with weight status and adiposity of school children after adjusting for different variables.

This study found that physical activity (> 2 times/week) had an independent inverse relationship with overweight/obesity and triceps skinfold thickness. Physical activity had a similar relationship with BMI-defined overweight/obesity in a sample of Pakistani primary school children [29]. Children in our study exhibiting low physical activity had significantly higher median triceps skinfold thickness compared to those with high physical activity. A report indicated that children who were moderately active were at risk of accumulating excess body fat compared to those who were active [35]. Also, a recent US study revealed that children who did not meet the physical activity guidelines were at risk of being overweight/obese [36]. It is a well-known fact that energy expenditure in children is increased when they are involved in physical activity, and apart from reducing the risk of overweight/obesity [8], it could also lead to a favorable body composition. However, a study in Nigeria did not record any association between physical activity and BMI [37]. In the Nigerian study, there could have been recall bias because physical activity was self-reported by the children and also, the study had a small number of overweight/obese participants. In our study, physical activity was reported by parents, and we cannot rule out the possibility of recall bias. However, this was limited by asking children to hand in the questionnaires only to their parents/guardians (who are presumed to be most familiar with the children and have a higher ability to recall their activity levels) to complete the questionnaires.

In the current study population, out of school sedentary lifestyle and occupation class were also independent predictors of overweight/obesity after controlling for age, gender, design variables and all variables that were significant in the bivariate analysis. When all the variables in the unadjusted analysis plus age and gender were included in quantile regression analysis, sedentary lifestyle and occupation class had the largest significant relationships with triceps skinfold thickness. A report on English children showed a positive association between sedentary behavior (which was questionnaire-based) and BMI. However, the authors observed no association between objectively measured sedentary behavior and BMI [38]. Also, a recent Cameroon study indicated that SES was significantly associated with BMI-defined overweight/obesity and central obesity in children [39]. A recent systematic review associated objectively and subjectively measured sedentary activities to unfavorable body composition [40]. The associations observed in our study could be explained by the fact that in Cameroon, high income earners can easily afford for items that, could contribute to a sedentary lifestyle to some extent like a TV set, computer and motorization to and from school. These items are readily available in urban than rural settings. During the study period, it was also observed that these items are common in private schools where fees are higher than public schools. Most children in some private schools in urban settings use bus services to school and spend their play time in school watching TV and these, to an extent, contribute to higher levels of sedentary lifestyle. However, we did not examine the independent contribution of these aspects to sedentary lifestyle. These children in private schools can therefore accumulate more body fat as a report had confirmed that children attending private schools had higher odds of not meeting physical activity guidelines [41].

This study has also shown that sedentary lifestyle and physical activity were associated with occupation class. A higher proportion of children with low physical activity and high sedentary lifestyle were in the high occupation class, while a higher proportion of children with high physical activity and low sedentary lifestyle were in the low occupation class. In Cameroon, low income earners and those in rural settings are usually involved in more long distance walking and they carry out high intensity activities like farming, which extends to children. Also, available data in Cameroon shows that daily energy expenditure is higher among rural subjects that urban subjects [5]. A report had associated sedentary lifestyle with living in an urban setting and high income background [42].

Parental level of education was not significantly associated with overweight/obesity in the bivariate analysis. Matsudo et al. [41] reported a similar observation among Brazilian children. Education level does not seem to be a good indicator of socioeconomic status in Cameroon as being educated does not assure a better remuneration as indicated in a previous study [32]. However, a significant inverse association between parental level of education and overweight/obesity was recorded in a US study [43]. The area of residence (rural and urban) and type of school (public and private) were not associated with overweight/obesity in our study. However, a recent systematic review revealed that urban residence may be positively associated with overweight/obesity in Sub-Saharan Africa [44]. Also, a study in Kenya (which included a convenient sample of school children) indicated that children attending private schools had higher odds of being overweight/obese [25]. These inconsistencies may be explained by small sample sizes of studies and differences in sampling procedures.

Our study had limitations worth mentioning. This study presents results of out of school physical activity levels in children, which were not assessed with respect to physical activity domains. For instance, when school hours was included in a recent report as one of the domains of objectively measured physical activity, it contributed to 55 and 46% of total sedentary and moderate-to-vigorous physical activity respectively [45]. The sample of children was not nationally representative and causal relationships cannot be established using a cross sectional design. It was also not possible to control for the confounding effect of puberty. In addition, parental occupation class was established using the Cameroon government classification system, which may not be similar to that of the private service. Further, the findings of this study should be interpreted with caution because calorie intake, which is an important determinant of body weight, was not assessed. A WHO report had indicated that the evidence showing a positive association between overweigh/obesity and the consumption of energy dense foods poor in micronutrients is convincing [46]. Other studies have indicated the association of low intakes of fruits and vegetables, large portion sizes [47] and high consumption of sugar-sweetened beverages [48] with changes in weight. But it is still a debate if reducing intake of sugar-sweetened beverages can lead to weight loss [49]. These suggest that the associations observed in this study could change if dietary intake and eating habits are included in the multivariable models.

Despite the above limitations, this study presents data on out of school physical activity, sedentary lifestyle and adiposity measured using triceps skinfold thickness among school children for the first time in Cameroon. The use of quantile regression in the analysis allowed us to determine the effect of the different factors on the median of triceps skinfold thickness. Even though the study uses proxy reporting, a review had indicated that proxy measures can predict physical activity levels in children [50] and may also complement objective measurements because they can provide information on the type of physical activity [44]. The data sets the basis for future studies, especially with initiatives aimed at establishing and validating an instrument that can be used to assess physical activity and sedentary lifestyle of children in our setting.

## Conclusion

This study has shown an inverse relationship between physical activity and overweight/obesity. It also shows that sedentary lifestyle and occupation class were positively associated with overweight/obesity and had the largest significant relationship with triceps skinfold thickness.

In order to understand the activity patterns of children in our setting, domain-specific physical activity and sedentary lifestyle need to be assessed objectively and subjectively, in and out of school. Also longitudinal studies are warranted to understand the influence of environmental, cultural and behavioral drivers of physical activity and sedentary lifestyle in our multiethnic setting, where fatness is still being regarded as a sign of good health and wellbeing. In addition, the school environment can significantly influence the development of obesity if renovations or adjustments are made to provide not just nutrition education, but also opportunities for physical activity and enough time for physical education especially in urban-private schools.

## Abbreviations

BMI: Body mass index
NWR: North West Region
TV: Television
WHO: World Health Organization
